# Cross-Cultural Adaptation and Validation of the Persian Version of the Oxford Knee Score in Patients with Knee Osteoarthritis

**Published:** 2014-11

**Authors:** Mohammad Hosein Ebrahimzadeh, Hadi Makhmalbaf, Ali Birjandinejad, Seyed Hosein Soltani-Moghaddas

**Affiliations:** Orthopedic Research Center, Ghaem Hospital, Mashhad University of Medical Sciences, Mashhad, Iran

**Keywords:** Iran, Knee, Surgery, Osteoarthritis, Knee replacement arthroplasty, Public health

## Abstract

**Background: **The Oxford Knee Score (OKS) is a short patient-reported outcome instrument that measures pain and physical activity related to knee osteoarthritis. The purpose of this study is to evaluate, construct validity and consistent reliability of the Persian version of the OKS.

**Methods: **The case series consisted of 80 patients who were clinically diagnosed with having knee osteoarthritis. All patients were requested to fill-in the Persian OKS and Short-Form 36 Health Survey (SF-36). Correlation analysis between the Persian versions of these two instruments was then carried out. The scores of the Persian SF-36 were used to evaluate convergent and divergent validity of the 12-item Persian OKS.

**Results: **From a total of 80 patients, 63 were female (79%) and the remaining 17 were male (21%) with a mean age of 52.2 years. In the present study, high Cronbach’s alpha of 0.95 confirms excellent internal consistency of the Persian OKS scale similar to previous investigations. The results confirm that the Persian version of this instrument is valid and reliable, similar to its English index and its subsequent translations in different languages.

**Conclusion: **The Persian OKS is a reliable instrument to evaluate knee function in patients with knee osteoarthritis and is a useful tool for outcome measurement in clinical research.

## Introduction

Osteoarthritis (OA) of the knee is the most prevalent major human joint disease and a leading cause of disability in the elderly. In addition, this musculoskeletal disorder affects health related quality of life worldwide. It is characterized by pain and physical disability in walking, sitting, standing and climbing stairs. It can affect all aspects of daily activity of a sufferer, such as self-care, work, social and leisure activities.^1^^-^^5^

Due to the high prevalence of knee OA in Asia, the Middle East and specifically in Iran,^6^ not only non-operative, but operative treatments and proper evaluation of results are important.^7^ The prevalence of symptomatic knee OA is as high as 15.3% in the urban and 19.3% in the rural areas of Iran.^8^ The goals of knee OA treatments are to improve daily living activities; health related quality of life and reduces pain and discomfort.

Internationally validated and standardized clinical outcome instruments to assess health and life quality of knee OA patients, allows investigators to compare clinical results across cultures, regions and countries. It has been demonstrated that the quality of life questionnaires or patient-oriented assessment instruments are more reliable since they are closely associated with the patient’s experience and health status.^2^

The Oxford Knee Score (OKS) is a short patient-reported outcome instrument that measures pain and physical activity related to the knee. It was first drafted and validated by Dawnson et al. in 1998 for knee joint replacement patients.^7^ Until now, it has been validated in several languages with good reliability, including French, Dutch, Japanese, German, Italian, Swedish, Thai and Portuguese.^2^^,^^4^^,^^9^^-^^14^

Considering the high prevalence of knee OA in our region and amongst the Persian speaking population of countries such as Iran, Afghanistan and Tajikistan, the OKS as an internationally recognized clinical measure needs to be validated in the Persian language (Farsi).

The objective of the present study was to formulate the OKS applicable for clinical studies of Persian speaking patients with knee OA by its translation, cross-cultural adaptability, and its validity and reliability evaluation. 

## Materials and Methods

*Translation and Cultural Adaptation*

*Oxford Knee Score*

**Figure 1:**
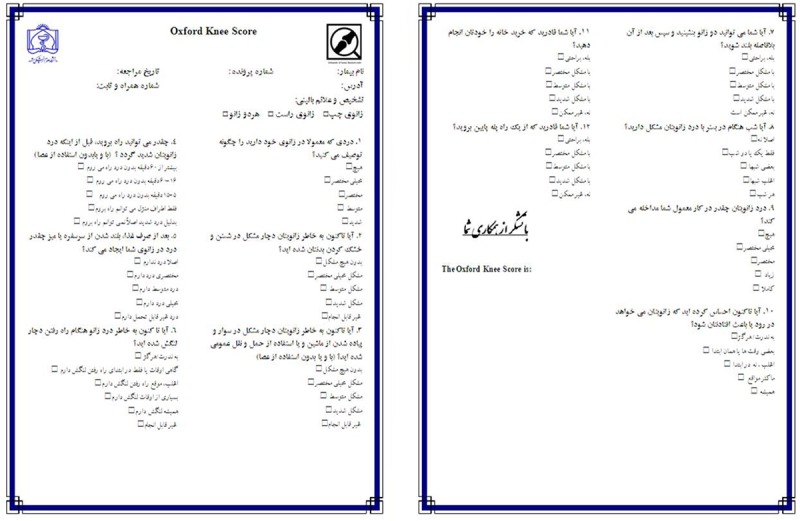
Persian Version of Oxford Knee Score

The Persian translation of the OKS was done in accordance with Guillimin et al. and Beaton et al.’s methodology for cultural adaptation of health related quality of life measures.^3^^,^^5^^,^^7^^,^^12^ Three bilingual translators (one professional English language translator and two orthopedic surgeons) translated the original English OKS into the Persian. In a panel meeting, the group, including the primary investigator, developed a draft version of the Persian OKS. As a cross check, backward translation into the English language was carried out by an English language translator (native English and fluent in Persian). To minimize bias, the latter translator was unaware of the original English version of the OKS questionnaire and its clinical application. In a final meeting of the involved parties, the concluding version of the Persian OKS was produced with minimal changes (figure 1).

The OKS is a questionnaire consisting of 12 items that cover pain and daily living activities related to the knee. It includes two subscales of pain and function, each with five categories of responses ranked on a Likert scale. It is recommended to score the questions from zero to four, with 48 as the maximum total score.^15^

*Short-Form 36 Health Survey (SF-36)*

The SF-36 health related survey is a well-known clinical outcome measure of health related quality of life and health status. It is a widely validated questionnaire for different cultures and in various languages around the world including Persian.^16^

This instrument measures health related quality of life on eight multi-item dimensions, including physical functioning, role physical, bodily pain, social functioning, role emotional, vitality, mental health and general health. 

*Patients*

This research was carried out in 2011 at the Knee and Shoulder Center of Ghaem Hospital of Mashhad University of Medical Sciences, Mashhad, Iran. The research proposal was approved by the research committee of the above-mentioned Institute. The consent forms were signed by the participants and they were debriefed about the study. The case series consisted of 80 patients who were diagnosed with primary knee OA by an orthopedic surgeon member of the research team, according to radiographic and clinical criteria of the American College of Rheumatology.^17^ All patients filled in the Persian versions of the OKS and SF-36 health survey forms. To crosscheck the reliability aspect, 30 randomly selected patients filled in the Persian OKS again a week later.

Inclusion criteria for patients were having knee primary OA being referred to our knee and shoulder clinic, native Persian and the ability to read and write in Persian. For the re-test sample patients, the inclusion criterion was not having received any major treatment such as knee replacement within the previous week. For each patient, the research team recorded the time it took to fill in the Persian OKS and occasionally clarified items on need basis.

*Tests*

*Validity*

Construct validity is the degree to which a test measures what it claims, or purposes, to be measured. Validity refers to a comparison of the current test with a formerly standardized test.

Correlation analysis between the Persian versions of the OKS with the SF-36 was calculated. The scores of the eight dimensions of the Persian SF-36 were utilized to evaluate convergence and divergence validity of the 12-item Persian OKS. Construct validation was calculated using the Pearson correlation coefficients between the 12-item Persian OKS and the Persian SF-36.

Our hypothesis was that, there would be a high correlation coefficient between the Persian OKS and the Persian SF-36 physical domains (bodily pain, role of physical limitation, physical functioning). Content validity was examined by evaluating the distribution, floor and ceiling effect of the 12 questions of the OKS. This content validity allows us to understand whether the Persian OKS items cover all domains of the patient. The ceiling effect is the highest possible score and floor effect is the lowest. For this purpose, the proportion of answer frequencies with the lowest possible score (0) and the highest possible score (48) was calculated. When more than 15% of the case series achieved the highest and lowest total scores, a floor and ceiling effect are considered.^1^^,^^18^

*Reliability (Internal Consistency)*

To evaluate reproducibility of the items of the Persian OKS, test-retest reliability, and Interclass Correlation Coefficient (ICC) was calculated. For this reason, we randomly asked 30 patients to fill in the 12-item OKS seven days later. During this period, they did not receive any major treatments that could substantially change their condition.

The ICC ranges from 0.00 (no agreement) to 1.00 (full agreement) describes the extent of answer repetition by the patients.

Internal consistency of the Persian OKS was evaluated by the Cronbach`s alpha; it indicates good reliability when this index is between 0.7 and 0.9.^18^

## Results

The participants included 80 patients with a diagnosis of OA of three compartments of the knee by standing X-ray. They were 63 (79%) women and 17 (21%) men with a mean age of 52.2 years. Demographics of the patients are summarized in table 1.

**Table 1:** Demographic Characteristic of the patients

**Age **	
Mean (SD)	52.2 (14.2)
Median (Min:Max)	(24:84)
Gender, N (%)	
Male	17 (21.2%)
Female	63 (78.8%)
Education, N (%)	
Below High school degree	53 (65.5%)
High school and up	27 (34.5%)
Diagnosis	Primary knee osteoarthritis

The average time for patients to fill in the questionnaire was 9±6, ranging from 7 to 18 minutes.

*Validity*

Construct validation test showed that there was a significant correlation between OKS with the six domains of the SF-36, including PF (r=0.63), RP (r=0.57), BP (r=0.82), GH (r=0.38), VT (r=0.42), SF (r=0.57), RE (r=0.27), PCS (r=0.75), (P<0.001) but there were insignificant correlation with MH (P=0.179) and MCS (P=0.51). These results are shown in table 2.

**Table 2:** Correlation between different items of the SF-36 and total score of the Persian OKS

	**Mean (SD) **	Correlation with OKS*
Sf-36 (PF)	36.3 (25.1)	R=0.639** P=0.000
Sf-36 (RP)	16.2 (31.0)	R=0.570** P=0.000
Sf-36 (BP)	29.8 (21.5)	R=0.829** P=0.000
Sf-36 (GH)	50 (21.3)	R=0.387** P=0.000
Sf-36 (VT)	53.3 (21.5)	R=0.426** P=0.000
Sf-36 (SF)	55.6 (29.6)	R=0.571** P=0.000
Sf-36 (RE)	50 (45.6)	R=0.271* P=0.015
Sf-36 (MH)	64.2 (22.4)	R=0.152 P=0.179
Sf-36 (PCS)	28.5 (9.57)	R=0.750** P=0.000
Sf-36 (MCS)	48.05 (11.6)	R=0.219 P=0.051

*Correlation is significant at the 0.05 level (2-tailed); **Correlation is significant at the 0.01 level (2-tailed); PF: Physical function; RP: Role physical; BP: Bodily pain; GH: General health; VT: Vitality; SF: Social function; RE: Role emotional; MH: Mental health; PCS: Physical composite score; MCS: Mental composites score; SD: Standard deviation; R: Pearson correlation coefficient

*Reliability*

In the present study, the Cronbach`s alpha of all items of the OKS questionnaire was calculated. Cronbach`s alpha, based on standardized items was 0.915; therefore, the reliability of the Persian version of the OKS is excellent. In this study, the calculated ICC with a 95% confidence interval was 0.95. The correlation between questions of the OKS shows that the majority of the questions are correlated. The results are summarized in table 3.

**Table 3:** Correlation between questions of OKS to calculate of reliability

		**Q1**	**Q2**	**Q3**	**Q4**	**Q5**	**Q6**	**Q7**	**Q8**	**Q9**	**Q10**	**Q11**
Q2	r	0.311**	1									
	p	0.005										
Q3	r	0.369**	0.610**	1								
	p	0.001	0.000									
Q4	r	0.270*	0.405**	0.577**	1							
	p	0.016	0.000	0.000								
Q5	r	0.527**	0.541**	0.530**	0.508**	1						
	p	0.000	0.000	0.000	0.000							
Q6	r	0.383**	0.504**	0.616**	0.495**	0.568**	1					
	p	0.000	0.000	0.000	0.000	0.000						
Q7	r	0.495**	0.456**	0.513**	0.486**	0.520**	0.361**	1				
	p	0.000	0.000	0.000	0.000	0.000	0.001					
Q8	r	0.345**	0.324**	0.158	0.279*	0.403**	0.260*	0.201	1			
	p	0.002	0.003	0.160	0.012	0.000	0.020	0.073				
Q9	r	0.350**	0.580**	0.518**	0.342**	0.381**	0.497**	0.485**	0.271*	1		
	p	0.001	0.000	0.000	0.002	0.000	0.000	0.000	0.015			
Q10	r	0.201	0.291**	0.410**	0.102	0.186	0.392**	0.109	0.072	0.220*	1	
	p	0.074	0.009	0.000	0.367	0.099	0.000	0.337	0.525	0.050		
Q11	r	0.287**	0.529**	0.695**	0.687**	0.568**	0.459**	0.635**	0.216	0.422**	0.212	1
	p	0.010	0.000	0.000	0.000	0.000	0.000	0.000	0.054	0.000	0.059	
Q12	r	0.359**	0.582**	0.569**	0.594**	0.437**	0.460**	0.569**	0.322**	0.494**	0.247*	0.624**
	p	0.001	0.000	0.000	0.000	0.000	0.000	0.000	0.004	0.000	0.027	0.000

**Correlation is significant at the 0.01 level (2-tailed); *Correlation is significant at the 0.05 level (2-tailed)

*Floor and Ceiling Effects*

Results show that floor effect was observed in questions 2, 4, and 10. Apart from this, item numbers 1, 7, 8, and 9 are shown to have a ceiling effect. In addition to these, we can see that questions 6 and 11 have ceiling and floor effects. The results are summarized in table 4 and 5.

**Table 4:** The interclass correlation coefficient (ICC) for questions of the Persian OKS

	**Mean (SD)**	**Floor effect (%)**	**Ceiling effect (%)**	**ICC**
1. How would you describe the pain you usually have in your knee?	3.2 (0.82)	1.2%	56.2%	0.91
2. Have you had any trouble washing and drying yourself (all over) because of your knee?	1.61 (1.25)	30%	1.2%	
3. Have you had any trouble getting in and out of the car or using public transport because of your knee? (With or without a stick)	2.08 (1.11)	13.8%	3.8%	
4. For how long are you able to walk before the pain in your knee becomes severe? (With or without a stick)	2.06 (1.31)	17.5%	13.8%	
5. After a meal (sat at a table), how painful has it been for you to stand up from a chair because of your knee?	2.11 (1.12)	8.8%	7.5%	
6. Have you been limping when walking, because of your knee?	2.11 (1.46)	17.5%	26.2%	
7. Could you kneel down and get up again afterwards?	3.22 (1.14)	6.2%	56.2%	
8. Are you troubled by pain in your knee at night in bed?	2.64 (1.23)	10%	32.5%	
9. How much has pain from your knee interfered with your usual work? (including housework)	2.72 (1.14)	6.2%	26.2%	
10. Have you felt that your knee might suddenly give away or let you down?	1.22 (1.21)	35%	3.8%	
11. Could you do household shopping on your own?	2.18 (1.48)	21.2%	22.5%	
12. Could you walk down a flight of stairs?	2.10 (1.10)	10%	5%	
Total	20.4 (9.8)	1.2%	1.2%	

**Table 5:** Comparison of validity studies of dimensions of the SF-36 and the OKS questionnaire in various articles in Persian, Portuguese, Dutch, Italian, Korean

**Short ** **form-36**	**Sf-36(PF)**	**Sf-36(RP)**	**Sf-36(BP)**	**Sf-36(GH)**	**Sf-36(VT)**	**Sf-36(SF)**	**Sf-36(RE)**	**Sf-36(MH)**	**Sf-36(MCS)**	**Sf-36(PCS)**
Persian OKS version	R=0.63 P=0.001	R=0.57 P=0.001	R=0.82 P=0.001	R=0.38 P=0.001	R=0.42 P=0.001	R=0.57 P=0.001	R=0.27 P=0.015	R=0.15 P=0.17	R=0.21 P=0.051	R=0.75 P=0.001
Portuguese OKS Version4	R=-0.71 P<0.001	R=-0.46 P<0.05	R=-0.77 P<0.001	R=-0.39 P<0.05	R=−0.28 P=0.05	R=−0.45 P<0.05	R=−0.33 P<0.05	R=−0.32 P<0.05	-----	----
Dutch OKS Version11	R=0.64 P<0.001	R=0.40 P<0.001	R=0.68 P<0.001	----	R=0.14 P<0.001	R=0.46 P<0.001	R=0.26 P<0.001	R=0.27 P=0.5	----	-----
Italian OKS version14	R=0.49 P<0.02	R=0.53 P<0.02	R=0.63 P<0.02	R=0.41 P<0.05	R=0.43 P<0.05	R=0.42 P<0.05	R=0.20 P>0.05	R=0.96 P<0.05	R=0.69 P<0.05	R=0.21 P>0.05
Korean OKS version20	R=0.66 P<0.01	R=0.49 P<0.01	R=0.30 P<0.01	R=0.11 P=0.16	R=0.15 P=0.06	R=0.48 P<0.01	R=0.30 P<0.01	R=0.15 P=0.04	-----	----

## Discussion

The high prevalence of knee OA in Iran and the growing interest for knee replacement reflects on the need for a disease specific “self-assessment knee outcome instrument” in the Persian language for clinical documentation and research. According to recommendations by Guillemin et al.,^5^ using a clinical measurement tool without validating the translated version can not suffice.

The aim of this study was to translate the site/disease specific 12-item patient-reported Oxford Knee Score into Persian with the focus on its validation. To our knowledge, this is the first study on the validation of the OKS among the Persian speaking population.

This version showed construct validity, reliability, internal consistency and content validity, similar to the original English OKS and its subsequent translated versions in other languages and cultures.^12^^,^^15^^,^^19^ The construct validity of the Persian version of the OKS was calculated by correlation between summed scores of the Persian OKS with the mean scores of different domains of the Persian SF-36 amongst our participants.

Table 5 summarizes validation studies of the OKS in the Persian, Portuguese, Dutch, Italian and Korean languages. Cronbach`s alpha obtained in the test-retest reliability in the Italian, Dutch, Portuguese, French, Swedish and Singaporean (English, Chinese and Korean), Korean versions were 0.90, 0.94, 0.87, 0.88, 0.93, 0.8 and 0.91 respectively.^4,9,11,18,20,21^ In the present study, the high Cronbach`s alpha coefficient (0.95) confirms the internal consistency of the OKS Persian version scale similar to the previous translations and cultural adaptations; therefore, we can conclude excellent reliability of the Persian OKS.

Regarding the ceiling effect in question 1, the probable cause could be due to the nature of the question as it inquires about pain. It is a fact that pain is a common finding in patients with knee problems and majority refer to clinics due to pain. In question 2, regarding “… trouble washing and drying yourself…” is a task which does not necessarily require a healthy knee. Most patients can sit on a chair and perform the task using their hands.

The floor effect in question 4 is possibly because of the type of patients that was included in our study. This question is dependent on the severity of the knee OA and patients with a lesser degree of OA have a better ability to walk.

Floor and ceiling effects in question 6 are due to the type of involvement in such patients. Most patients with OA would not limp until late stages of the disease. 

Regarding questions 7, 8, and 9, possible cause of the ceiling effect could be due to the type of question, which specifically focuses on the knee. For question 7, as kneeling down followed by standing up could put a great amount of pressure on the knee, most of the patients gave it a high score. For the next question, as mentioned before, pain is a common finding in OA patients. In question 9, as the knee joint plays an important role in the movement of the body, more than 15% of the participants believed that their knee pain had a high amount of interference with their usual routine. 

Generally, in patients with OA, we rarely observe sudden giving way, unless they are in the late stages of the disease.^22^Therefore, in question 10, a floor effect of 35% can be observed.

A possible explanation for the floor and ceiling effects in question 11 is due to the fact that complicated tasks such as shopping, requires an entirely healthy knee since this task involves kneeling, walking and other forms of activities. High correlation and level of significance between the Persian OKS with the SF-36 domains indicate excellent validity of our Persian version of the OKS. 

## Conclusion

The results of this study confirm that the Persian version of the OKS is valid and reliable, similar to its English index and its subsequent translation in French, Dutch, Italian, and other languages. Therefore, the Persian OKS is a reliable instrument to evaluate knee function in patients with knee OA and is a useful tool for outcome measurement in clinical research. 
